# Pain medicine content, teaching and assessment in medical school curricula in Australia and New Zealand

**DOI:** 10.1186/s12909-018-1204-4

**Published:** 2018-05-11

**Authors:** Elspeth Erica Shipton, Frank Bate, Raymond Garrick, Carole Steketee, Eric John Visser

**Affiliations:** 1 School of Medicine, University of Notre Dame Australia, UNDA, P O Box 1225, Fremantle, WA 6160 Australia; 2 School of Medicine, University of Notre Dame Australia, UNDA, Sydney, Darlinghurst Campus, Darlinghurst, NSW 2000 Australia; 3 The Learning and Teaching Office, University of Notre Dame Australia, UNDA, P O Box 1225, Fremantle, WA 6160 Australia

**Keywords:** Pain medicine, education, curricula, medical student

## Abstract

**Background:**

The objective of pain medicine education is to provide medical students with opportunities to develop their knowledge, skills and professional attitudes that will lead to their becoming safe, capable, and compassionate medical practitioners who are able to meet the healthcare needs of persons in pain. This study was undertaken to identify and describe the delivery of pain medicine education at medical schools in Australia and New Zealand.

**Method:**

All 23 medical schools in Australia and New Zealand in 2016 were included in this study. A structured curriculum audit tool was used to obtain information on pain medicine curricula including content, delivery, teaching and assessment methods.

**Results:**

Nineteen medical schools (83%) completed the curriculum audit. Neurophysiology, clinical assessment, analgesia use and multidimensional aspects of pain medicine were covered by most medical schools. Specific learning objectives for pain medicine were not identified by 42% of medical schools. One medical school offered a dedicated pain medicine module delivered over 1 week. Pain medicine teaching was delivered at all schools by a number of different departments throughout the curriculum. Interprofessional learning (IPL) in the context of pain medicine education was not specified by any of the medical schools. The mean time allocated for pain medicine teaching over the entire medical course was just under 20 h. The objective structured clinical examination (OSCE) was used by 32% of schools to assess knowledge and skills in pain medicine. 16% of schools were unsure of whether any assessment of pain medicine education took place.

**Conclusion:**

This descriptive study provides important baseline information for pain medicine education at medical schools in Australia and New Zealand. Medical schools do not have well-documented or comprehensive pain curricula that are delivered and assessed using pedagogically-sound approaches considering the complexity of the topic, the prevalence and public health burden of pain.

**Electronic supplementary material:**

The online version of this article (10.1186/s12909-018-1204-4) contains supplementary material, which is available to authorized users.

## Background

Pain medicine is a discipline within the field of medicine that is concerned with the prevention of pain, and the evaluation, treatment, and rehabilitation of persons in pain [1]. Medical practitioners, along with other health professionals, play an essential role in the management of acute and chronic cancer and non-cancer pain [2]. Acute pain is one of the most common reasons why patients seek treatment at an emergency department, and remains a common problem in the post-operative setting [3–6] . In a recent Australian study, 47% of patients experienced moderate-to-severe pain 1 week after surgery [7]. Pain is common, but often poorly managed following cancer treatment with an estimated prevalence rate for certain types of cancer from 29% to 57% [8, 9]. Epidemiological studies in Australia and New Zealand reported that approximately 20–25% of the adult population experience moderate-to-severe chronic pain [10–15]. The incidence of chronic pain can also be higher in at-risk groups such as the young and the elderly. Community studies have shown that pain is prevalent in children and adolescents, with median prevalence rates ranging from 11 to 38% [16]. An Australian study of community-dwelling older adults showed prevalence rates of 32–62% experiencing pain [17]. The prevalence of chronic pain in Australia is projected to increase as the population ages (from around 3.2 million in 2007 to 5.0 million by 2050) [18]. Chronic pain has been suggested as the largest unmet clinical problem in Australia and New Zealand [19]. The Global Burden of Disease Study 2013 placed back and neck pain as the leading cause of disability-adjusted life years in Australia and New Zealand, ahead of ischaemic heart disease, depression, chronic obstructive pulmonary disease, Alzheimer’s, lung cancer, stroke and diabetes [19]. Excellent evidence based guidelines for the management of acute and chronic pain are available [20, 21].

Pain continues to be undiagnosed and undertreated, resulting in considerable cost to individuals and society [18, 22]. Fewer than 10% of patients with chronic non-cancer pain gain access to effective care, although 80% could be treated effectively by applying current best-practice management [23]. In Australia, more than a quarter of patients referred to a chronic pain management service remained on waiting lists for more than a year [24]. Hospitals vary widely in the provision of acute and chronic pain management services and many interdisciplinary pain clinics in the tertiary sector are understaffed [25, 26]. Pain Medicine is a relatively new speciality in Australia and New Zealand, and there are currently not enough qualified Specialist Pain Medicine Physicians (SPMPs) to service the entire population [27, 28]. There may never be enough specialist resources to meet the needs of patients with chronic pain and it is therefore essential that the emphasis is also placed on greater capacity for treatment of acute and chronic pain in the primary care setting [29]. Primary care providers (PCPs) have reported inadequate training regarding pain management, limited confidence in their ability to provide effective pain treatment, uncertainty over prescribing opioids for chronic pain and a low level of satisfaction with their care of chronic pain patients [30–37]. In a recent large study in Europe, most PCPs perceived chronic pain to be one of the most challenging conditions to treat [38].

The lack of education and training in the discipline of pain medicine among health professionals has been highlighted as one of the barriers to best-practice pain management [22, 39, 40]. Recent research shows that there is a paucity of pain-related content in most medical school curricula internationally [39, 41–43]. Introducing change into a fixed-calendar medical curriculum is difficult as this requires existing items being replaced. However, rather than adjusting existing curricula to slot in new material, a more holistic approach to teaching pain medicine could be considered – one that encourages course and curriculum coordinators to consider the changing societal needs that underpin the importance of training health professionals in the discipline of pain medicine.

Educators recognise that the process of curriculum change needs to be deliberately and purposefully managed in order to accommodate these changing healthcare requirements in society whilst maintaining the fundamental standards and values of the institution, [44]. Lee et al. (2013) argue that the Four Dimensional Framework for Curriculum Development (4DF) offers a mechanism by which the multi-dimensional and often complex nature of health professional curriculum can be examined and developed [44]. This framework is comprised of four dimensions that alert educators and curriculum developers to the local, societal and political issues that should be considered when developing curriculum. These four dimensions are i) future healthcare practice needs, ii) competencies and capabilities required of graduates, iii) teaching, learning and assessment methods, and iv) institutional parametres. As stated by Lee et al. (2013, p.69), each of these dimensions “conveys a message about issues that matter, for example, what will be known, done, why and how and by whom, how its effects will be measured and its impacts evaluated” [44]. Such a tool might be useful for conceptualising the priorities, as well as the constraints, associated with pain medicine curriculum design and integration. No gold standard has been specified as to what pain content is necessary in an ideal medical curriculum. However, recent attention has been focussed on the development of pain-focussed curricula in order to connect international scientific knowledge with expertise and practice; the most utilised of these is the core curriculum developed by the International Association for the Study of Pain (IASP) [2, 42, 45–55]. This research was undertaken to examine existing pain medicine education curricula at medical schools in Australia and New Zealand so as to identify areas that are adequately covered, as well as providing an understanding of the strengths and limitations of current curricula.

In Australia, the National Pain Strategy (NPS) launched in Canberra in 2010 was developed by the National Pain Summit Initiative led by the Faculty of Pain Medicine (FPM) of the Australian and New Zealand College of Anaesthetists (ANZCA), the Australian Pain Society and Chronic Pain Australia in collaboration with the MBF Foundation (now Bupa Foundation) and the University of Sydney Pain Management Research Institute [56]. This was the first comprehensive initiative in Australia, which aimed to improve the quality of life for people with pain and their families, and to minimise the burden of pain on individuals and the community [56]. The NPS highlighted the need for high priority to be given to the development of a national pain management curriculum for medical students; the designation of pain management as a key competency in undergraduate and postgraduate education for the medical workforce; and the training and support of health practitioners in best-practice pain assessment and management (especially in the biopsychosocial processes underpinning acute and chronic pain) [56]. No formal evaluation has been undertaken to assess whether these NPS objectives have been met.

The FPM is the professional body responsible for the education, training and continuing professional development of SPMPs in Australia and New Zealand [57]. The strategic vision of the FPM is “to reduce the burden of pain in society through education, advocacy, training and research” [57]. Whilst training and education of specialist pain medicine physicians remains the primary focus of the FPM’s role, education of medical students is also seen as an important function [57].

The FPM distributed a document entitled “Pain and the Undergraduate Medical Curriculum” to all medical schools and training organisations in Australia and New Zealand in 2010 [58]. Thirteen learning objectives for medical students pertaining to pain medicine were identified and a framework was included of how to achieve these learning objectives. Details of comprehensive pain medicine curricula were included [46, 59]. Assistance was offered in terms of the development of pain medicine curricula with the provision of educational resources; information on recommended minimum standards for graduating medical students regarding knowledge of pain medicine; information about the availability of accredited pain medicine training units for access by medical schools for the training of medical students; as well as a list of SPMPs who could be approached to teach aspects of pain medicine [58]. It also encouraged the inclusion of a suitable SPMPs on the curriculum committee to assist with integration of pain medicine principles into the medical curriculum [58]. Whether or not the medical schools implemented the recommendations outlined in the document in 2010 is unknown.

There is currently limited literature on pain medicine education for medical students across Australasia. Accordingly, this study was undertaken to identify and describe the delivery of pain medicine education at medical schools in Australia and New Zealand. Specifically, the study sought to develop understandings around the following aspects of pain medicine curricula:

- What is taught (topic areas, learning outcomes)
- How it is packaged (stand-alone modules or integrated; interprofessional approach; resources; electives; as well as the proportion of time devoted to it in the program)
- How it is sequenced (at what stage(s) in the course, and how often it is addressed)
- Who teaches it (specialist pain medicine educators or educators from a range of medical disciplines)
- How it is taught (e.g. lecture, small group teaching, problem-based learning)
- What and how it is assessed (multiple choice questions, short answer questions, objective structured clinical examination (OSCE))

## Methods

This descriptive, exploratory study included all 23 schools of medicine in Australia and New Zealand training medical students in 2016. Information on the current pain curricula (major topics in pain) taught within these medical schools was gathered using a curriculum audit tool. The curriculum audit tool was developed by the authors from a review of current pain and medical education literature [2, 41–44, 48, 49, 51, 60, 61]. In particular, the 4DF and the IASP curriculum for medical students were used to formulate the specific questions included in the audit tool [2, 44]. With regards to the 4DF, the four dimensions were used as a guide to ensure questions in the audit were comprehensive and addressed both local and broader issues related to the development of pain medicine curriculum (e.g., who is responsible for conceptualizing the pain medicine curriculum?; which pain medicine framework has been adopted for guiding content?; what teaching and assessment methods have been used to deliver the curriculum?; and what are the university nuances that have shaped the design and delivery of the pain medicine curriculum?). Seventeen key topics in pain medicine were identified from the IASP core curriculum (see Additional file 1). Ten experts in the disciplines of medical education or clinical pain medicine revised questions for content and face-validity. Ethics approval was obtained from the University of Notre Dame Australia.

The curriculum audit took place between October 2016 and April 2017. An information letter was emailed to the medical school Deans of Education or their delegates requesting permission to access information on aspects of the curriculum relating to pain medicine. A representative was then identified by the Dean (or delegate) as someone who had a detailed knowledge about pain education in the curriculum. In the event the Dean could not identify a representative to complete the curriculum audit, the researcher approached a person in the school who was either co-ordinating the pain education curriculum, or who had detailed knowledge of it in the curriculum (e.g., an FPM fellow or lecturer in pain education). A letter along with the curriculum audit tool was sent to each representative including an explanation of the study, and an assurance of confidentiality. The representative was encouraged to corroborate data with colleagues and students. Non-respondents received reminder emails at regular intervals. The completed curriculum audit tool was scanned and returned by email. The data was de-identified so that no medical school could be identified during the analysis of the data.

Numeric and descriptive data were obtained and statistical analysis was conducted using SPSS software (see Additional file 1 for curriculum audit tool scoring schedule). Blank items were coded as missing and excluded from analysis. Descriptive statistics were used to present frequencies and percentages of pain education content in medical schools’ curricula. Where appropriate, measures of central tendency and variability were calculated. The sample size (*n* = 19) was insufficient for further analysis such as tests for statistical significance. Since the study was explorative and descriptive in nature, this was not seen as a concern.

## Results

Nineteen of the 23 medical schools provided information, reflecting an 83% response rate for the curriculum audit tool. Some questions allowed multiple responses and, as a result, the percentages did not always total 100%.

### Demographic characteristics of the sample

64% of respondents indicated that they had collaborated with other educators or students to provide the required information. Of the thirteen respondents who identified themselves as being from the medical profession, ten were SPMPs from various disciplines including anaesthetics, psychiatry, neurology and general practice. Overall, nine of respondents had an anaesthetic qualification. Table 1 below summarises the key demographic characteristics of the respondents.

**Table 1:** Demographic characteristics of respondents

Characteristic	n	%
Gender		
Male	12	63.2
Female	7	36.8
Professional Status		
Medical	13	68.4
Allied Health Practitioner	3	15.8
Non-Clinical Educators	3	15.8

### Pain-related content or topics covered in the medical curriculum

As evident in Table 2, neurophysiology of pain (100%), clinical assessment (95%), analgesia use (95%) and the multidimensional model of pain medicine (90%) were covered by most medical schools. Adjuvant analgesics, palliative/cancer pain and the concept of peripheral/central sensitisation were each taught by 68% of schools. Fewer than half the schools covered the topic of psychological methods for managing pain, medical interventions and ethics. The multidisciplinary pain clinic (26%), medico-legal aspects of pain medicine (21%), geriatric pain (21%) and paediatric pain (21%) were topics covered by the least number of schools.

**Table 2:** Frequencies of pain-related content or topics covered in the medical curriculum

Pain Related Topics	N	%
Neurophysiology	19	100.0
Clinical Assessment	18	94.7
Primary Analgesics	18	94.7
Multi–dimensional Model of Pain	17	89.5
Central and Peripheral Sensitisation	13	68.4
Secondary Analgesics	13	68.4
Palliative care	13	68.4
Aetiology	12	63.2
Physiotherapy Management	11	57.9
Acute Pain Team	10	52.6
Psychological Management	9	47.4
Medical Interventions	8	42.1
Ethics	6	31.6
Multidisciplinary Pain Clinic	5	26.3
Medicolegal aspects	4	21.1
Paediatric Pain	4	21.1
Geriatric Pain	4	21.1
Other	3	15.8

*Note. Percentages are based on number of responses and do not total 100%*

*N, number of medical schools for which elements of the curriculum were available*

The IASP core curriculum was partially implemented in 42% of medical schools. No school had fully implemented the IASP core curriculum. 26% of schools indicated that they were unsure whether the IASP curriculum had been implemented or not, whilst 32% had not implemented the IASP curriculum at all.

### Specified Learning Objectives (SLO) related to Pain Medicine

SLOs were not identified or were unknown at 42% of medical schools. The following learning objectives were identified by medical schools: clinical assessment of a patient in pain (58%), neurophysiology of pain (53%), analgesics (47%) and the multidimensional model of pain (42%) (see Table 3 below).

**Table 3:** Frequencies of reported specified learning objectives

Specified Learning Objectives	N	%
Clinical Assessment	11	57.9
Neurophysiology	10	52.6
Primary Analgesics	9	47.4
Multi-dimensional Model of Pain	8	42.1
Psychological Management	6	31.6
Aetiology	5	26.3
Central/Peripheral Sensitisation	4	21.1
Medical Interventions	4	21.1
Physiotherapy Management	4	21.1
Secondary Analgesics	4	21.1
Palliative Care	3	15.8
Ethics	3	15.8
Other	2	10.5
Clinical Exposure Acute Pain Team	2	10.5
Medicolegal	1	5.3
Paediatric Pain	1	5.3
Geriatric Pain	1	5.3
No Specified Learning Objectives	8	42.1

*Note. Percentages are based on number of responses and do not total 100%*

*N, number of medical schools for which elements of the curriculum were available*

### Integrated or Stand-alone pain module

The pain medicine curriculum was taught as a specified (stand-alone) module at one medical school (5%). This 24-h module was delivered during a one-week period and was compulsory for all students. This school also delivered further pain medicine education within other subject areas throughout the medical course. In 95% of schools, pain medicine was taught as a topic integrated into other compulsory subject areas. This teaching was spread over the entire curriculum.

The pain medicine curriculum was delivered mainly from the Departments of Anaesthesia (74%), Physiology/Neurophysiology (58%) and Pharmacology (47%), as shown in Table 4. A number of other departments were identified across the different medical schools.

**Table 4:** Frequencies of departments delivering pain medicine content in the curriculum

Departments delivering pain medicine content	N	%
Department(s) delivering pain medicine content		
Anaesthesiology	14	73.7
Physiology	11	57.9
Pharmacology	9	47.4
Palliative Care	7	36.8
Orthopaedics	6	31.6
Psychology	5	26.3
Clinical Skills	5	26.3
General Practice	4	21.1
Anatomy	4	21.1
Rheumatology	3	15.8
General Surgery	3	15.8
Internal Medicine	3	15.8
Obstetrics/Gynaecology	3	15.8
Geriatrics	2	10.5
Neurology	2	10.5
Psychiatry	2	10.5
Paediatrics	2	10.5
Intensive Care	2	10.5
Microbiology/Biochemistry	1	5.3
Pathology	1	5.3
Rehabilitation	1	5.3
Advanced Learning	1	5.3
Emergency	1	5.3
Ethics	1	5.3
Pain Medicine	1	5.3
Health Economics	1	5.3

*Note. Percentages are based on number of responses and do not total 100%*

*N, number of medical schools for which elements of the curriculum were available*

### Time allocated to pain medicine

Time allocated for pain medicine teaching during the entire medical curriculum, ranged from 5 to 43 h, with a mean of 19.6 h (SD = 10.9).

### Elective

53% of schools offered a student elective in pain management ranging from 2 to 6 weeks.

### Pain education resources

37% of schools indicated they did not use any specific pain education resources such as pain medicine text books, e-learning modules or shared education programmes. Of those schools that used specific pain education resources, 32% used books, and 26% used the four-hour basic pain medicine education module, Essential Pain Medicine (EPM). 11% of schools reported using the National Prescribing Service pharmacy e-learning module and a further 16% used undisclosed e-learning tools.

### Interprofessional learning (IPL)

IPL involves creating opportunities for students from a range of health professional courses to learn with, from and about each other [62, 63]. 79% of medical schools indicated that medical students were not exposed to IPL in the context of pain medicine education. The remaining 21% were unsure whether IPL occurred within their institution.

### Professionals and disciplines delivering pain medicine education

With specific regard to availability of specialists or recognised experts in the field of pain medicine to assist with the pain medicine education, 90% of medical schools indicated that there were SPMP’s involved in the teaching of medical students, 37% of schools involved specialist physiotherapists, 37% employed psychologists and 32% engaged nurses, as evident in Table 5.

**Table 5:** Frequencies for specialists/recognised experts in the field of pain medicine within medical schools

Specialist	N	%
SPMP	17	89.5
Physiotherapist	7	36.8
Psychologist	7	36.8
Nurse	6	31.6
Occupational Therapist	3	15.8
Anaesthetist	2	10.5
Palliative Care	2	10.5
Psychiatrist	1	5.3
Non-clinical scientist	1	5.3
Rheumatologist	1	5.3
No	1	5.3
Unsure	1	5.3

*Note. Percentages are based on number of responses and do not total 100%*

*N, number of medical schools for which elements of the curriculum were available*

Medical clinicians delivered pain medicine teaching at all 19 medical schools, alongside non-clinical lecturers in almost half the schools. 37% of medical schools involved allied health personnel in the delivery of the pain medicine curriculum (see Table 6).

**Table 6:** Frequencies for categories of professionals delivering pain medicine content in the curriculum

Professional delivering pain content	N	%
Medical Clinician	17	100.0
University Lecturer (non-clinical)	7	52.6
Allied Personnel	7	36.8
Simulation Instructor	6	5.3

*N, number of medical schools for which elements of the curriculum were available*

### Teaching methods

All medical schools used didactic teaching methods. Clinical exposure was frequently included as a teaching method (84%). 47% of schools used tutorial teaching methods and 42% adopted case-based learning (See Additional file 2 for Glossary). Problem–based learning was used by 26% schools and e-learning by 21% of schools. Self-directed learning and simulation based learning were used infrequently (See Table 7 below).

**Table 7:** Frequencies of reported teaching methods

Teaching Method	N	%
Didactic Learning	19	100.0
Clinical Exposure	16	84.2
Tutorial	9	47.4
Case Based Learning	8	42.1
Problem Based Learning	5	26.3
E-Learning	4	21.1
Self-Directed Learning	3	15.8
Simulation Based Learning	2	10.5

*Note. Percentages are based on number of responses and do not total 100%*

*N, number of medical schools for which elements of the curriculum were available*

### Assessment methods

As shown in Table 8, multiple choice questions (MCQs) were used as an assessment tool for pain medicine education by 63% of schools and the objective structured clinical examination (OSCE) was used by 32% of schools. 16% of schools were unsure of whether any assessment of pain medicine education took place.

**Table 8:** Frequencies of reported assessment methods

Assessment Methods	N	%
MCQ	12	63.2
Short Answer	9	47.4
Case Based Report	9	47.4
OSCE	6	31.6
Assignment	2	10.5
Online	1	5.3
Observed	1	5.3
Integrated Performance Assessment	1	5.3
Unsure/ Not	3	15.8

*Note. Percentages are based on number of responses and do not total 100%*

*N, number of medical schools for which elements of the curriculum were available*

## Discussion

Whilst pain medicine is included in the curricula of medical schools in Australia and New Zealand, in general, these schools do not have well-documented or comprehensive pain medicine curricula that are taught using pedagogic approaches that accommodate the complexity of the topic. There is little evaluation of pain medicine competencies as a requirement for graduation. Most medical schools indicated that it was a difficult task to access specific details of information regarding the pain medicine curricula required for this curriculum audit project. Reasons for this included a lack of communication between educators in preclinical and clinical years, the varying nature of pain medicine teaching from year to year, and the curriculum delivery dependent on the presence of an enthusiastic clinical pain champion on the staff.

### Pain medicine curricula

There appears to be wide variations in the range of pain medicine topics covered by the medical schools. It is encouraging to note the incorporation of aspects of the biopsychosocial model of pain medicine into the curriculum (e.g. the inclusion of a topic on the multidimensional nature of pain medicine at 90% of schools). Recent advances in the understanding of pain (e.g. the introduction of concepts such as central sensitisation at 68% of schools) have also emerged in the curriculum. Topics such as psychological methods for managing pain, ethics and medico-legal aspects of pain medicine, the multidisciplinary pain clinic, geriatric pain and paediatric pain are neglected. These topics are intrinsic to the recommended pain medicine curricula proposed by internal leading experts that have been available for many years [2]. Students need to be provided not only with the necessary clinical knowledge of pain medicine, but also prepared to address the serious professional, personal, and ethical challenges that arise in caring for those with pain [64]. Similar lack of attention to these key subjects has been identified at the majority of medical schools in North America [43] . The lack of teaching about the multidisciplinary pain clinic has previously been identified at medical schools in Finland [53]. The curriculum audit found that the current focus of the curriculum is on medical treatment of pain primarily using analgesics. This finding is concerning as it detracts from the interdisciplinary approach to chronic non-cancer pain management that has scientific evidence for efficacy [65, 66]. Secondly, it focuses on the traditional goal emphasizing the restoration of health or cure, which is not realistic in many instances when managing patients with pain. Pain management, especially, chronic pain management, is more about reducing symptoms, improving function, understanding the patient’s biological, psychological and social experience of pain, and empathy rather than offering a ‘cure’ [40, 67].

Defining explicit objectives are the basis of modern curriculum design to identify and align elements of the curriculum such as content, learning experiences, teaching strategies and assessment [68]. Core competencies in pain assessment and management for medical students have been developed by leading interprofessional health care clinicians and educators [69]. These core competencies are consistent with the domain outline from the IASP pain curricula [69]. This study has shown that specific pain medicine learning objectives are not identifiable at 42% of universities, and are incomplete at most medical schools. Whilst topics such as neurophysiology, clinical assessment, and primary analgesics were included in the curriculae of most medical schools, learning objectives for these topics were only specified by less than 60% of medical schools. This finding is problematic as without clearly articulated learning objectives, educators do not know what to teach. The ‘what’ of pain medicine is left to chance and open to bias in terms of what individuals think students should know, rather than what the formal curriculum states they need to know. In the light of ‘constructive alignment’ [70], which reasons that assessment should be firmly aligned to clearly specified learning objectives, one could infer that in curriculum where content is not linked to learning objectives, there is no formal mechanism for ensuring that what is intended to be learned, has been taught accordingly, nor assessed.

In general, pain medicine was not taught as a distinct stand-alone module. One medical school (5%) had a dedicated compulsory pain module consisting of 24 h teaching spread over a one-week period. This finding resonates with other research in the USA, which reports that 4% of schools offered a compulsory pain course, and the duration of these courses ranged from 1.5 to 13 days [43].

Most universities in Australia and New Zealand have an integrated model where there is a synchronous, trans-disciplinary delivery of information between the foundational sciences and the applied sciences throughout all years of a medical school curriculum [71]. Whilst no optimal model of delivery of pain medicine education has been identified, pain curricula can be successfully incorporated into the integrated model when delivered in a planned, comprehensive and measurable manner [41]. According to the present audit, 95% of schools taught pain medicine as a topic integrated into other compulsory subject areas over the entire curriculum. Pain was mostly taught in anaesthesia, neurophysiology, pharmacology and palliative care modules, a similar finding to other studies of pain medicine education at medical schools in Europe [41, 53]. The mean number of departments involved in pain medicine education was five. There was evidence of a lack of co-ordinated planning for pain medicine education between departments or between clinical/non-clinical years at each of the 19 medical schools. There was no mechanism in place to ensure that the core elements of the topic were addressed and integrated into different subject areas. This lack of coordination is likely to result in a fragmented, ineffectual understanding of pain [53], with pain, particularly chronic pain, being seen as a symptom of other conditions rather than a disease entity per se [72, 73].

The sustainability of anaesthetists continuing to teach pain medicine is under threat. Firstly, a recent study to develop curriculum priorities for the teaching of anaesthesia and anaesthetic topics to medical students in Australia and New Zealand revealed that whilst acute pain was still considered an essential topic to be included in an ideal curriculum, chronic pain was not [74]. Secondly, the ANZCA revised curriculum (2013) specifically excluded a three-month pain medicine module (Module 10) that was previously part of the mandatory registrar rotation. As a result, today’s anaesthetic registrars are not receiving a foundational understanding of the biopsychosocial model of pain medicine, nor in chronic pain medicine [75]. There is also a real possibility that pain medicine is under exposed due to the limited representation of SPMPs as heads of anaesthetic departments as well as on curriculum planning committees, similar to findings in other countries [40, 64].

Students who show a particular interest in pain medicine are able to undertake an elective in pain management at 53% of universities. This is a positive finding and one which contrasts with other literature. For example, in the USA, only 16% of medical schools offered a designated pain elective [43].

A mean of 19.6 h (median of 20 h) is allocated for pain medicine teaching during the entire medical curriculum at medical schools in Australia and New Zealand. This commitment equates to approximately 0.3% of the minimum total teaching hours for the undergraduate medical degrees in New Zealand (approximately 7900 h) and 0.4% of a post-graduate medical degree in Australia (approximately 5640 h). The proportion of time allocated to pain medicine is comparable to countries in Europe (median of 12 h allocated for compulsory pain courses), the USA (mean of 11 h), the UK (mean of 13 h), and Canada (mean of 28 h) [41–43, 60] allocated to pain teaching at medical schools. In 2011, 20% of medical schools in the USA still appeared to have less than five hours of teaching on the topic [43]. These findings point to limited attention being paid to pain medicine in the medical curriculum both locally and internationally considering the clinical and societal burden of pain disease.

### Teaching and Assessment Methods

IPL has been shown to be effective for improving medical students’ pain competencies in a variety of settings, including general, paediatric and acute pain management [54, 76–78]. However, medical schools in this study did not identify IPL as a delivery strategy for pain medicine education.

Pain education and training is best provided by specialists (medical and allied) uniformly and reliably trained in pain medicine [79, 80]. It is encouraging, therefore, to note that the majority of universities in the current study indicated that they had staff who are specialists or recognised experts in the field of pain medicine to assist with the teaching of pain medicine to medical students. However, only approximately a third of universities had specialist physiotherapists, psychologists and nurses delivering pain education. There appears to be a lack of educational activity to prepare healthcare students for collaborative pain management, despite the recognition that pain is best managed in a multidisciplinary setting [41].

Following international trends, medical schools in Australia and New Zealand have adopted more student-centred approaches to teaching and learning in medical courses [81, 82]. Although this study shows that lectures remain the most common teaching method for pain medicine, the medical schools generally employed a few different teaching methods at each institution. There is evidence to show that pain education needs to address both the affective and cognitive dimensions of pain [48]. Innovative teaching methods such as narrative writing, role playing, fine art and motivational interviewing have shown to be successful for teaching medical students about the affective dimensions of pain whilst fostering positive emotional development of students [83, 84]. Pain medicine education in Australia and New Zealand currently does not focus on developing emotional skills and reflective capacity, similar to the situation at medical schools in the USA and Europe [41, 43, 64].

There appeared to be limited use of specific pain resources for teaching pain medicine, especially web-based resources. A similar finding has been identified in Europe and the UK [41, 42]. The incorporation of an e-learning resource such as the one developed at the Virginia Commonwealth University could be considered by medical schools to disseminate pain content in a flexible, modular approach [49]. Sharing of such resources would spare medical schools the expense of creating their own resources, and instead allow them to put more effort into adapting the resources to meet local needs.

There is no national licensing examination in Australia and New Zealand, so medical schools have their own assessment processes to ensure that graduates are prepared for internship [82]. Final-year medical students are not specifically required to display adequate knowledge and skills regarding pain management as a requirement for graduation.

This study has shown that, when undertaken, assessment methods pertaining to pain medicine are predominantly MCQ’s, short-answer questions and case-based reports. These methods are unlikely to assess skill-based pain competencies and attitudes. The OSCE-type assessment was used only in a third of medical schools in this study to assess pain competencies. 16% of medical schools lacked any pain-focused assessment. This study did not find compelling evidence to suggest that graduate medical students in Australia and New Zealand possess adequate competencies in pain management.

### Strengths and Limitations

The present study has a number of limitations. Detailed information regarding the medical school curricula was difficult to obtain. The lack of documentation of pain medicine in the curriculum itself indicates the low priority given to this essential aspect of medical education. The medical curriculum is not publically available in New Zealand and Australia, as it is in other countries [41]. More recent studies have used only web-based information to examine pain medicine education [41, 72]. However, many universities in Australia and New Zealand are only in the developmental stages of using web-based curriculum maps to outline specific details of learning objectives, lecture content and delivery, and assessment methods. The use of a specially designed curriculum audit as used in this study has been employed previously and most, like the one used in this study, have been based on the IASP core curriculum [42, 53, 60, 61]. The data collected represents the perceptions of a limited number of individuals and these perceptions may differ from those of the broader academic community. However, a key strength of this study was the recruitment of an interested participant who was active in the teaching of pain management at each medical school. In most cases, this person liaised with other educators involved with the teaching of pain medicine at the medical school. This data collection strategy reduced the non-response rate and provided a more accurate, nuanced and comprehensive overview of the pain medicine teaching at each institution. The findings cannot be generalised to the medical schools that did not participate in the study. However, 83% of medical schools provided information which can be considered a representative sample. The 4DF was an appropriate and useful tool to structure this research into pain medicine curriculum. It has proven to be an effective tool by different individuals and institutions for review and development of curricula and curriculum redesign [85]. Further qualitative research involving a variety of stakeholders is being undertaken to obtain a broader understanding of the strengths and limitations of pain medicine education in Australia and New Zealand.

## Conclusion

This descriptive study provides important baseline information for pain medicine education at medical schools in Australia and New Zealand. Medical schools do not have well-documented comprehensive pain curricula that are delivered and assessed using modern pedagogically-sound approaches considering the complexity of the topic, the prevalence and public health burden of pain. Pain medicine education in New Zealand and Australia is limited, variable and fragmented, similar to findings in Europe and the USA [41, 43]. Pain-related learning objectives, when specified, do not reflect the broad topic as recommended by the IASP and students are not required to display competencies in pain medicine for graduation. Multidisciplinary pain management (especially psychological pain management), ethics and medico-legal aspects of pain treatment, as well as paediatric and geriatric pain appear to be underrepresented in medical curricula. It seems that calls for curriculum reform by leading professional organisations such as the NPS and FPM have not been heeded.

Health care providers need to be equipped with the necessary knowledge, attitudes and skills to meet the professional and ethical challenges that arise in caring for those in pain, and so to manifest both competence and compassion toward their patients. A more holistic approach to teaching pain medicine should be considered. A flexible modular approach that can be integrated over the entire medical curriculum may be the best way to structure the pain curricula for some universities (as implemented at the University of Washington), or a week long in-depth course such as is offered at the University of Toronto or John Hopkins University may fit better with other medical schools [48, 51, 54]. A framework tool would be useful for conceptualising the priorities, as well as the constraints, associated with pain medicine curriculum design and for purposeful management of the complex process of curriculum change [44].

## Additional files

Additional file 1: Medical School Pain Curriculum Audit Scoring Tool, This audit scoring tool was used to gather information on the pain curricula at medical schools. Details of this audit tool are presented. (DOCX 14 kb)

Additional file 2: Glossary, Definition of terms. (DOCX 18 kb)

## Abbreviations

4DF: Four Dimensional Framework for Curriculum Development
ANZCA: Australian and New Zealand College of Anaesthetists
EPM: Essential Pain Medicine
FPM: Faculty of Pain Medicine
IASP: International Association for the Study of Pain
IPL: Interprofessional learning
MCQs: Multiple choice questions
NPS: National Pain Strategy
OSCE: Objective Structured Clinical Examination
PCPs: Primary care providers
SLO: Specified learning objectives
SPMPs: Specialist Pain Medicine Physicians
UK: United Kingdom
USA: United States of America

## References

[1] American Board of Pain Medicine. Definition of Pain Medicine http://www.abpm.org/what. Accessed 20 July 2017.

[2] International Association for the Study of Pain. http://www.iasp-pain.org/Education/CurriculumDetail.aspx?ItemNumber=729 Accessed 4 July 2014.

[3] Carr EC, Meredith P, Chumbley G, Killen R, Prytherch DR, Smith GB (2014). Pain: a quality of care issue during patients’ admission to hospital. J Adv Nurs.

[4] Motov SM, Khan AN (2008). Problems and barriers of pain management in the emergency department: Are we ever going to get better?. J Pain Res.

[5] Apfelbaum JL, Chen C, Mehta SS, Gan TJ (2003). Postoperative pain experience: results from a national survey suggest postoperative pain continues to be undermanaged. Anesth Analg.

[6] Institute of Medicine. Relieving pain in America: A blueprint for transforming prevention, care, education, and research. Washingtin, DC: National Academies Press; 2011. 22553896

[7] Veal FC, Thompson AJ, Perry LJ, Bereznicki LR, Peterson GM (2018). Pain intensity and pain self-management strategies following discharge after surgery: An Australian prospective observational study. J Clin Pharm Ther.

[8] Ilhan E, Chee E, Hush J, Moloney N (2017). The prevalence of neuropathic pain is high after treatment for breast cancer: a systematic review. Pain.

[9] Boland EG, Ahmedzai SH (2017). Persistent pain in cancer survivors. Curr Opin Support Palliat Care.

[10] Breivik H, Collett B, Ventafridda V, Cohen R, Gallacher D (2006). Survey of chronic pain in Europe: prevalence, impact on daily life, and treatment. Eur J Pain.

[11] Hoy D, Bain C, Williams G, March L, Brooks P, Blyth F (2012). A systematic review of the global prevalence of low back pain. Arthritis Rheum.

[12] Schopflocher D, Taenzer P, Jovey R (2011). The prevalence of chronic pain in Canada. Pain Res Manag.

[13] Dominick CH, Blyth FM, Nicholas MK (2012). Unpacking the burden: understanding the relationships between chronic pain and comorbidity in the general population. Pain.

[14] Tsang A, Von Korff M, Lee S, Alonso J, Karam E, Angermeyer MC (2008). Common chronic pain conditions in developed and developing countries: gender and age differences and comorbidity with depression-anxiety disorders. J Pain.

[15] Blyth FM, March LM, Brnabic AJ, Jorm LR, Williamson M, Cousins MJ (2001). Chronic pain in Australia: a prevalence study. Pain.

[16] King S, Chambers CT, Huguet A, MacNevin RC, McGrath PJ, Parker L (2011). The epidemiology of chronic pain in children and adolescents revisited: a systematic review. Pain.

[17] Laslett LL, Quinn SJ, Winzenberg TM, Sanderson K, Cicuttini F, Jones G (2012). A prospective study of the impact of musculoskeletal pain and radiographic osteoarthritis on health related quality of life in community dwelling older people. BMC Musculoskelet Disord.

[18] MBF Foundation. The high price of pain: the economic impact of persistent pain in Australia: MBF Foundation; 2007. https://www.bupa.com.au/staticfiles/BupaP3/Health%20and%20Wellness/MediaFiles/PDFs/MBF_Foundation_the_price_of_pain.pdf. Accessed 1 May 2018.

[19] Murray CJ, Barber RM, Foreman KJ, Abbasoglu Ozgoren A, Abd-Allah F, Abera SF (2015). Global, regional, and national disability-adjusted life years (DALYs) for 306 diseases and injuries and healthy life expectancy (HALE) for 188 countries, 1990–2013: quantifying the epidemiological transition. Lancet.

[20] Schug S, Palmer G, Scott D, Halliwell R, Trinca J (2015). Acute Pain Management: Scientific Evidence.

[21] Management ASoATFoCP. American Society of Regional Anesthesia and Pain Medicine (2010). Practice guidelines for chronic pain management: an updated report by the American Society of Anesthesiologists Task Force on Chronic Pain Management and the American Society of Regional Anesthesia and Pain Medicine. Anesthesiology.

[22] Kress HG (2013). The importance of putting pain on the curricula in medical schools in Europe. J Pain Palliat Care Pharmacother.

[23] Painaustralia. National Pain Strategy http://www.painaustralia.org.au/improving-policy/national-pain-strategy. Accessed 06 Apr 2018.

[24] Hogg MN, Gibson S, Helou A, DeGabriele J, Farrell MJ (2012). Waiting in pain: a systematic investigation into the provision of persistent pain services in Australia. Med J Aust.

[25] Stieg RL (2000). Financing the Treatment of Chronic Pain: Models for Risk-sharing among Pain Medicine Physicians, Health Care Payers, and Consumers. Pain Med.

[26] Mailis-Gagnon A, Yegneswaran B, Lakha S, Nicholson K, Steiman AJ, Ng D (2007). Pain characteristics and demographics of patients attending a university-affiliated pain clinic in Toronto, Ontario. Pain Res Manag.

[27] Shipton EA (2012). Recognition of the vocational practice of the scope of Pain Medicine in New Zealand. N Z Med J.

[28] Painaustralia. Painful Facts http://www.painaustralia.org.au/about-pain/painful-facts. Accessed 18 Aug 2017.

[29] Ellis B, Johnson M, Taylor A (2012). Education as part of wider health policy and improvement strategies. Br J Pain.

[30] Upshur CC, Luckmann RS, Savageau JA (2006). Primary care provider concerns about management of chronic pain in community clinic populations. J Gen Intern Med.

[31] Sinatra R (2006). Opioid analgesics in primary care: challenges and new advances in the management of noncancer pain. J Am Board Fam Med.

[32] Potter M, Schafer S, Gonzalez-Mendez E, Gjeltema K, Lopez A, Wu J (2001). Opioids for chronic nonmalignant pain. Attitudes and practices of primary care physicians in the UCSF/Stanford Collaborative Research Network. University of California, San Francisco. J Fam Pract.

[33] Ponte CD, Johnson-Tribino J (2005). Attitudes and knowledge about pain: an assessment of West Virginia family physicians. Fam Med.

[34] Bendtsen P, Hensing G, Ebeling C, Schedin A (1999). What are the qualities of dilemmas experienced when prescribing opioids in general practice?. Pain.

[35] Moulin DE, Clark AJ, Speechley M, Morley-Forster PK (2002). Chronic pain in Canada--prevalence, treatment, impact and the role of opioid analgesia. Pain Res Manag.

[36] Olsen Y, Daumit GL (2002). Chronic pain and narcotics: a dilemma for primary care. J Gen Intern Med.

[37] Clark JD (2002). Chronic pain prevalence and analgesic prescribing in a general medical population. J Pain Symptom Manag.

[38] Johnson M, Collett B, Castro-Lopes JM (2013). The challenges of pain management in primary care: a pan-European survey. J Pain Res.

[39] Doorenbos AZ, Gordon DB, Tauben D, Palisoc J, Drangsholt M, Lindhorst T (2013). A blueprint of pain curriculum across prelicensure health sciences programs: one NIH Pain Consortium Center of Excellence in Pain Education (CoEPE) experience. J Pain.

[40] Loeser JD (2015). The Education of Pain Physicians. Pain Med.

[41] Briggs EV, Battelli D, Gordon D, Kopf A, Ribeiro S, Puig MM (2015). Current pain education within undergraduate medical studies across Europe: Advancing the Provision of Pain Education and Learning (APPEAL) study. BMJ Open.

[42] Briggs EV, Carr EC, Whittaker MS (2011). Survey of undergraduate pain curricula for healthcare professionals in the United Kingdom. Eur J Pain.

[43] Mezei L, Murinson BB (2011). Johns Hopkins Pain Curriculum Development T. Pain education in North American medical schools. J Pain.

[44] Lee A, Steketee C, Rogers G, Moran M (2013). Towards a theoretical framework for curriculum development in health professional education. Focus Health Prof Educ.

[45] Argyra E, Siafaka I, Moutzouri A, Papadopoulos V, Rekatsina M, Vadalouca A (2015). How Does an Undergraduate Pain Course Influence Future Physicians’ Awareness of Chronic Pain Concepts?. Comp Stud Pain Med.

[46] European Federation of IASP Chapters. The Pain Management Core Curriculum for Undergraduate Medical Education http://www.europeanpainfederation.eu/wp-content/uploads/2016/12/CoreCurriculumPainManagement-EFIC-June-2013_FINAL.pdf. Accessed 1 May 2018.

[47] Charlton E (2005). Core curriculum for professional education.

[48] Murinson BB, Nenortas E, Mayer RS, Mezei L, Kozachik S, Nesbit S (2011). A new program in pain medicine for medical students: integrating core curriculum knowledge with emotional and reflective development. Pain Med.

[49] Yanni LM, Priestley JW, Schlesinger JB, Ketchum JM, Johnson BA, Harrington SE (2009). Development of a comprehensive e-learning resource in pain management. Pain Med.

[50] Stevens DL, King D, Laponis R, Hanley K, Zabar S, Kalet AL (2009). Medical students retain pain assessment and management skills long after an experiential curriculum: a controlled study. Pain.

[51] Tauben DJ, Loeser JD (2013). Pain education at the University of Washington School of Medicine. J Pain.

[52] Snyder R, Arwood S. Model Core Curriculum on Pain Management for Michigan Medical Schoolsm Michigan: Department of Licencing and Regulatory Affairs, Bureau of Health Services, State of Michigan; 2013 http://www.michigan.gov/documents/lara/Curriculum_MODEL_CORE_FINAL_APRIL_2013_MAILING_424376_7.pdf. Accessed 28 Sept 2015.

[53] Pöyhiä R, Niemi-Murola L, Kalso E (2005). The outcome of pain related undergraduate teaching in Finnish medical faculties. Pain.

[54] Hunter J, Watt-Watson J, McGillion M, Raman-Wilms L, Cockburn L, Lax L (2008). An interfaculty pain curriculum: lessons learned from six years experience. Pain.

[55] Watt-Watson J, Hunter J, Pennefather P, Librach L, Raman-Wilms L, Schreiber M (2004). An integrated undergraduate pain curriculum, based on IASP curricula, for six health science faculties. Pain.

[56] Lippe PM, Brock C, David J, Crossno R, Gitlow S (2010). The First National Pain Medicine Summit--final summary report. Pain Med.

[57] Faculty of Pain Medicine ANZCA. About FPM 2017 http://fpm.anzca.edu.au/about-fpm. Accessed 18 May 2017.

[58] Faculty of Pain Medicine ANZCA. FPM Education Committee Report 2008 http://fpm.anzca.edu.au/communications/synapse-e-newsletter/synapse/issue-29-2007. Accessed 18 May 2017.

[59] Pilowsky I (1988). An outline curriculum on pain for medical schools. Pain.

[60] Watt-Watson J, McGillion M, Hunter J, Choiniere M, Clark A, Dewar A (2009). A survey of prelicensure pain curricula in health science faculties in Canadian universities. Pain Res Manag.

[61] Pöyhiä R, Kalso E (1999). Pain related undergraduate teaching in medical faculties in Finland. Pain.

[62] Barr H, Koppel I, Reeves S, Hammick M, Freeth DS. Effective interprofessional education: argument, assumption and evidence (promoting partnership for health). Oxford: Blackwell Publishing; 2005.

[63] Oandasan I, Reeves S (2005). Key elements for interprofessional education. Part 1: the learner, the educator and the learning context. J Interprof Care.

[64] Murinson BB, Gordin V, Flynn S, Driver LC, Gallagher RM, Grabois M (2013). Recommendations for a new curriculum in pain medicine for medical students: toward a career distinguished by competence and compassion. Pain Med.

[65] Flor H, Turk DC (2011). Chronic Pain: An Intergrated Biobehavioural Approach 1st ed.

[66] Gatchel RJ, Okifuji A (2006). Evidence-based scientific data documenting the treatment and cost-effectiveness of comprehensive pain programs for chronic nonmalignant pain. J Pain.

[67] Bair MJ (2011). Learning from our learners: implications for pain management education in medical schools. Pain Med.

[68] Tyler RW (1949). Basic principles of curriculum and instruction.

[69] Fishman SM, Young HM, Lucas Arwood E, Chou R, Herr K, Murinson BB (2013). Core competencies for pain management: results of an interprofessional consensus summit. Pain Med.

[70] Biggs J (2014). Constructive alignment in university teaching. Rev High Educ.

[71] Brauer DG, Ferguson KJ (2015). The integrated curriculum in medical education: AMEE Guide No. 96. Med Teach.

[72] Bradshaw YS, Patel Wacks N, Perez-Tamayo A, Myers B, Obionwu C, Lee RA (2017). Deconstructing One Medical School's Pain Curriculum: I. Content Analysis. Pain Med.

[73] Siddall PJ, Cousins MJ (2004). Persistent pain as a disease entity: implications for clinical management. Anesth Analg.

[74] Overton MJ, Smith N (2015). Anaesthesia priorities for Australian and New Zealand medical school curricula: a Delphi consensus of academic anaesthetists. Anaesth Intensive Care.

[75] Australian and New Zealand College of Anaesthetists. ANZCA training program Curriculum 2013 http://www.anzca.edu.au/documents/curriculum-appendix-one.pdf. Accessed 19 May 201f7.

[76] Salam T, Saylor JL, Cowperthwait AL (2015). Attitudes of Nurse and Physician trainees towards an interprofessional simulated education experience on Pain Assessment and Management. J Interprofessional Care..

[77] Hunter JP, Stinson J, Campbell F, Stevens B, Wagner SJ, Simmons B (2015). A novel pain interprofessional education strategy for trainees: assessing impact on interprofessional competencies and pediatric pain knowledge. Pain Res Manag.

[78] Hadjistavropoulos HD, Juckes K, Dirkse D, Cuddington C, Walker K, Bruno P (2015). Student evaluations of an interprofessional education experience in pain management. J Interprofessional Care.

[79] Gallagher RM (2002). Pain education and training: progress or paralysis?. Pain Med.

[80] Watt-Watson J, Murinson B (2013). Current challenges in pain education. Pain Manage.

[81] McKimm J, Wilkinson T, Poole P, Bagg W (2010). The current state of undergraduate medical education in New Zealand. Med Teach..

[82] Prideaux D (2009). Medical education in Australia: much has changed but what remains?. Med Teach..

[83] Murinson BB, Agarwal AK, Haythornthwaite JA (2008). Cognitive expertise, emotional development, and reflective capacity: clinical skills for improved pain care. J Pain.

[84] Mishra S (2015). Do we need to change the medical curriculum: regarding the pain of others. Indian Heart J.

[85] Moran MC, Steketee C, Forman D, Dunston R (2015). Using a research-informed interprofessional curriculum framework to guide reflection and future planning of interprofessional education in a multi-site context. J Res Interprof Pract Educ.

