# Supplementary material for: Pain medicine content, teaching and assessment in medical school curricula in Australia and New Zealand
Source: BMC Med Educ. 2018 May 11;18:110. doi: 10.1186/s12909-018-1204-4 (PMC5948674; doi:10.1186/s12909-018-1204-4)
Supplement: Supplementary file 1 — Medical School Pain Curriculum Audit Scoring Tool, This audit scoring tool was used to gather information on the pain curricula at medical schools. Details of this audit tool are presented. (DOCX 14 kb) [file 12909_2018_1204_MOESM1_ESM.docx]

**Medical School Pain Curriculum Audit Scoring Tool**

General questions:

1. Person responsible for ensuring that pain medicine is included in the curriculum at your medical school (Role at medical school and discipline)
2. Aware of any recommendations by local or international pain specialists for core competencies in pain medicine for medical students? (Yes – indicate which, No, Unsure)
3. International Association for the Study of Pain (IASP) core curriculum been implemented for your medical students? ( Fully; Partially; Not implemented; Considered but rejected; Don’t know)
4. Medical students share pain medicine content/modules with other non-medical health-care students? (Yes; No; Unsure; yes, please specify which disciplines are involved such as Dentistry, Nursing, Occupational Therapy, or Physiotherapy) and briefly describe how many hours are shared and method of teaching (e.g., shared lectures, interdisciplinary problem-based learning group, ward rounds, clinics)
5. School of Medicine have staff who are specialists or recognised experts in the field of pain medicine, to assist with the teaching of pain medicine to medical students? (Yes; No; Unsure; If yes, please specify field, e.g., Specialist Pain Medicine Physician; Pain Management Nurse Practitioner; Specialist Pain Physiotherapist; Specialist Pain Psychologist.)
6. Specific pain education resources (Yes – specify e.g. EMP (lite), e-modules, text books; No; Unsure)
7. Elective opportunities in pain management? (Yes; No; Unsure; If yes, specify for how long and through which department)

For each major topic in pain:

1. Learning Objectives as specified in medical curriculum
2. Time allocated (hours or minutes. If one lecture covers a number of topics, please divide the hour by the number of topics covered.)
3. Pain medicine taught as a stand-alone pain module (yes, in part, no)
4. Department or speciality responsible for teaching this content? (Which department or other discipline such as anaesthesia, medicine, anatomy)
5. Personnel delivering the subject content (e.g. Medical specialist, Medical Registrar, Registered Nurse, Physiotherapist, University lecturer)
6. Compulsory teaching (if not, please indicate which category of students would NOT receive the teaching)
7. Type of teaching method (Didactic lecture (DL), Problem-based learning (PBL), Simulation-based learning (SBL), Team-based learning (TBL), Case-based learning (CBL); Clinical experiences (CE), e-learning (EL), or other (specify))
8. Assessment method (Multiple choice questions (MCQ), Short answer questions, Observed Structured Clinical Examination(OSCE), Case-based reports, other –specify)

Major Topics in Pain (examples of content)

1. Neurophysiology/pain mechanisms (Types of pain –eg neuropathic, nociceptive, Nociception, Perception, Modulation)
2. Neurophysiology of chronic pain (Peripheral/Central Sensitization)
3. Aetiology/prevalence of Pain (Incidence, Causes, Disability, Economics)
4. Clinical Assessment( Examination of patient with pain, clinical presentation of Chronic/Acute pain, Interviewing a patient with pain)
5. Multidimensional nature of pain (Subjective/Objective Interpretation of pain, Understanding the biopsychosocial aspects of pain, patients’ pain

beliefs, meaning of pain)

1. Management with Primary Analgesics ( Placebo, Opioids, NSAID’s, COX inhibitors, Lignocaine, Risk assessment and Monitoring)
2. Management with Adjuvant Analgesics ( Tricyclics, SSRI, SNRI, Anti-epileptics specifically used for pain relief )
3. Medical Management Interventions ( nerve blocks, injections, neuromodulation for pain relief)
4. Non-Medical Management Psychological (specific therapies for pain management, sleep/ mood/ anxiety therapy, goal setting, CBT, Hypnosis,

Mindfulness)

1. Non-Medical Management Physiotherapy (Specific therapy pain management, Graded Motor Imagery, TENS, Acupuncture,

Hydrotherapy, Exercise, Soft tissue mobilisation)

1. Ethics of Pain Management(the right to pain management, Therapeutic relationship of patient and health professional, Challenges of managing

chronic pain patients, Self-evaluation of students’ own attitudes to patients with pain

1. Clinical Practice in Pain Medicine ( Exposure to an acute pain service)
2. Clinical Practice in Pain Medicine ( Exposure to a multidisciplinary pain clinic, Exposure to Rehabilitation/follow-up planning)
3. Medico legal aspects of Pain Management(Including: Substance abuse, Medico legal requirements, Sickness benefits)
4. Pain management of special groups of patients: Paediatrics
5. Pain management of special groups of patients: Geriatrics
6. Pain management of special groups of patients: Cancer/Palliative care
