# Supplementary material for: Pain medicine content, teaching and assessment in medical school curricula in Australia and New Zealand
Source: BMC Med Educ. 2018 May 11;18:110. doi: 10.1186/s12909-018-1204-4 (PMC5948674; doi:10.1186/s12909-018-1204-4)
Supplement: Supplementary file 2 — Glossary, Definition of terms. (DOCX 18 kb) [file 12909_2018_1204_MOESM2_ESM.docx]

**Glossary**

Case-based learning: active learning strategy in which students read and discuss complex, real-life scenarios

Case-based report: a detailed report of the symptoms, signs, diagnosis, treatment, and follow-up of an individual patient, often including a literature search on the topic.

Curriculum: The term curriculum usually implies a planned sequence of instruction in a specific field and often provides a statement of the desired pupil outcomes in terms of skills, performances, attitudes, and values as well as some description of the pedagogical approaches and assessment methods, and resources aligned to the course.

Elective: This is a period of time spent by the medical student usually in their final year of study in a clinical setting of their choice. It provides an opportunity for medical students to develop their skills by observing and participating in an area of medicine, often in a different country or unfamiliar setting, in order to broaden the scope of their exposure to clinical medicine.

e-learning: virtual learning experience using electronic technologies to access educational curriculum.

Entry-level medical education programme: A tertiary level course of study undertaken at a medical school providing a basic grounding in the core areas of medicine and physiology required for undertaking a practical period of training in order to gain entry to the medical profession[1].This could be an undergraduate or post-graduate course

Four Dimensional Curriculum Framework (4DF): 4DF is a tool that can be used to assist health educators structure research into curriculum development in health professional education and link educational practice to health policy and professional practice[2]. It is useful for understanding the entirety of the medical curriculum as well as defining and addressing the complexities of curriculum development.

Integrated curriculum: Synchronous, trans-disciplinary delivery of information between the foundational sciences and the applied sciences throughout all years of a medical school curriculum”[3].

Integrated Performance Assessment: a classroom-based assessment model that can be used for evaluating student’s communication (interpersonal, interpretive, and presentational).

Interprofessional learning: This involves opportunities for students from a range of health professional courses to learn with, from and about each other [4, 5].

Medical student: Undergraduate and graduate-entry students enrolled in a university degree leading to the qualification of medical practitioner.

Multiprofessional learning: Learning by students alongside each other to integrate the teaching within their own professional-specific model[4, 5].

Objective Structured Clinical Examination: a tool for evaluation, often used in the health sciences, to test clinical competency and skills such as communication, clinical examination, and medical procedures.

Pain education resources: these could include pain medicine text books, e-modules, or courses such as the EMP (lite)[6] .

Pain medicine: Pain medicine is a discipline within the field of medicine that is concerned with the prevention of pain, and the evaluation, treatment, and rehabilitation of persons in pain[7].

Pain management: This is the modulation of the subjective unpleasant sensory and emotional experience of pain. It encompasses the knowledge, attitudes and skills for the diagnosis and treatment of pain. It includes the basic sciences of pain processing, clinical presentation of different types of pain, differences between acute and chronic pain, multidimensional aspects of the pain experience, pharmacological and non-pharmacological pain treatment, ethics of pain treatment, specific therapeutic challenges.

Pain medicine education: teaching and learning associated with the acquisition of knowledge, skills, behaviours, and professional attitudes related to the management of persons experiencing pain

Problem-based learning: the use of appropriate problems to increase knowledge and understanding, involving independent study with subsequent group discussion[8].

Simulation-based learning: educational activity that utilizes simulation aides or standardized patients to replicate clinical scenarios.

Additional file references:

1. General Medical Council. Glossary -Acceptable overseas medical qualifications <http://www.gmc-uk.org/doctors/registration_applications/14392.asp> Accessed 20 July 2017.

2. Lee A, Steketee C, Rogers G, Moran M. Towards a theoretical framework for curriculum development in health professional education. Focus on Health Professional Education. 2013;14(3):70-83.

3. Brauer DG, Ferguson KJ. The integrated curriculum in medical education: AMEE Guide No. 96. Medical teacher. 2015;37(4):312-22.

4. Barr H, Koppel I, Reeves S, Hammick M, Freeth DS. Effective interprofessional education: argument, assumption and evidence (promoting partnership for health): John Wiley & Sons; 2008.

5. Oandasan I, Reeves S. Key elements for interprofessional education. Part 1: the learner, the educator and the learning context. Journal of interprofessional care. 2005;19 Suppl 1:21-38.

6. Goucke CR, Jackson T, Morriss W, Royle J. Essential Pain Management: An Educational Program for Health Care Workers. World journal of surgery. 2014.

7. American Board of Pain Medicine. Definition of Pain Medicine <http://www.abpm.org/what> Accessed 20 July 2017.

8. Wood DF. ABC of learning and teaching in medicine: Problem based learning. BMJ: British Medical Journal. 2003;326(7384):328.
